# An 18F‐FDG‐PET/CT‐based radiomics signature for estimating malignance probability of solitary pulmonary nodule

**DOI:** 10.1111/crj.13751

**Published:** 2024-05-09

**Authors:** Jingchi Zheng, Yue Hao, Yan Guo, Ming Du, Pengyuan Wang, Jun Xin

**Affiliations:** ^1^ Radiology Department Shengjing Hospital of China Medical University Shenyang China; ^2^ GE Healthcare Shenyang China; ^3^ Nuclear Medicine Department Shengjing Hospital of China Medical University Shenyang China

**Keywords:** CT, lung cancer, PET, Radiomics, solitary pulmonary nodules

## Abstract

**Background:**

Some solitary pulmonary nodules (SPNs) as early manifestations of lung cancer, it is difficult to determine its nature, which brings great trouble to clinical diagnosis and treatment. Radiomics can deeply explore the essence of images and provide clinical decision support for clinicians. The purpose of our study was to explore the effect of positron emission tomography (PET) with 2‐deoxy‐2‐[fluorine‐18] fluoro‐d‐glucose integrated with computed tomography (CT; ^18^F‐FDG‐PET/CT) combined with radiomics for predicting probability of malignancy of SPNs.

**Methods:**

We retrospectively enrolled 190 patients with SPNs confirmed by pathology from January 2013 to December 2019 in our hospital. SPNs were benign in 69 patients and malignant in 121 patients. Patients were randomly divided into a training or testing group at a ratio of 7:3. Three‐dimensional regions of interest (ROIs) were manually outlined on PET and CT images, and radiomics features were extracted. Synthetic minority oversampling technique (SMOTE) method was used to balance benign and malignant samples to a ratio of 1:1. In the training group, least absolute shrinkage and selection operator (LASSO) regression analyses and Spearman correlation analyses were used to select the strongest radiomics features. Three models including PET model, CT model, and joint model were constructed using multivariate logistic regression analysis. Receiver operating characteristic (ROC) curves, calibration curves, and decision curves were plotted to evaluate diagnostic efficiency, calibration degree, and clinical usefulness of all models in training and testing groups.

**Results:**

The estimative effectiveness of the joint model was superior to the CT or PET model alone in the training and testing groups. For the joint model, CT model, and PET model, area under the ROC curve was 0.929, 0.819, 0.833 in the training group, and 0.844, 0.759, 0.748 in the testing group, respectively. Calibration and decision curves showed good fit and clinical usefulness for the joint model in both training and testing groups.

**Conclusion:**

Radiomics models constructed by combining PET and CT radiomics features are valuable for distinguishing benign and malignant SPNs. The combined effect is superior to qualitative diagnoses with CT or PET radiomics models alone.

## Graphical abstract

PET combined with CT radiomics model is superior to CT or PET alone in predicting the malignance probability of solitary pulmonary nodules (SPNs). Combining the anatomical structure information of CT with the physiological and biochemical information of PET had a positive role in the judgment of SPN.

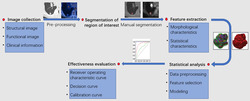

## 1 INTRODUCTION

Solitary pulmonary nodules (SPNs) are defined as a single nodule with a diameter <3 cm, round or oval shadows, clear or unclear boundaries, completely surrounded by normal lung parenchyma, and accompanied by no other abnormalities in the lung.^1^ While the majority are benign, around 35% are primary malignant tumors.^2^ When SPNs constitute the early manifestation of lung cancer, timely detection and appropriate treatment produces the highest cure rate, resulting in a 5‐year survival rate for those with IA stage as high as 80%.^3^ Surgery is the best treatment for malignant pulmonary nodules, but surgery should be avoided in patients with benign nodules. Correct diagnosis of SPNs can therefore assist clinical decision‐making, which is critical for the survival and prognosis of patients.

In clinical practice, pulmonary nodules are mainly diagnosed by conventional computed tomography (CT), enhanced CT, and positron emission tomography (PET) with 2‐deoxy‐2‐[fluorine‐18]fluoro‐D‐glucose integrated with CT (^18^F‐FDG‐PET/CT). According to these intuitive morphological characteristics, clinicians evaluate benign and malignant nodules based on long‐term accumulated experience, which is easily affected by subjective factors.^4^, ^5^ Generally, nodules with maximum standardized uptake value (SUVmax) > 2.5 tend to be diagnosed as malignant.^6^ However, many studies have reported false positive results in lung diseases where the intake of FDG is high, including granuloma, pneumonia, and in particular, tuberculosis.^7^ In another meta‐study, moderate accuracy was shown with PET/CT for the differential diagnosis of benign and malignant pulmonary nodules, although reliability still needed further improvement.^8^

**FIGURE 1:**
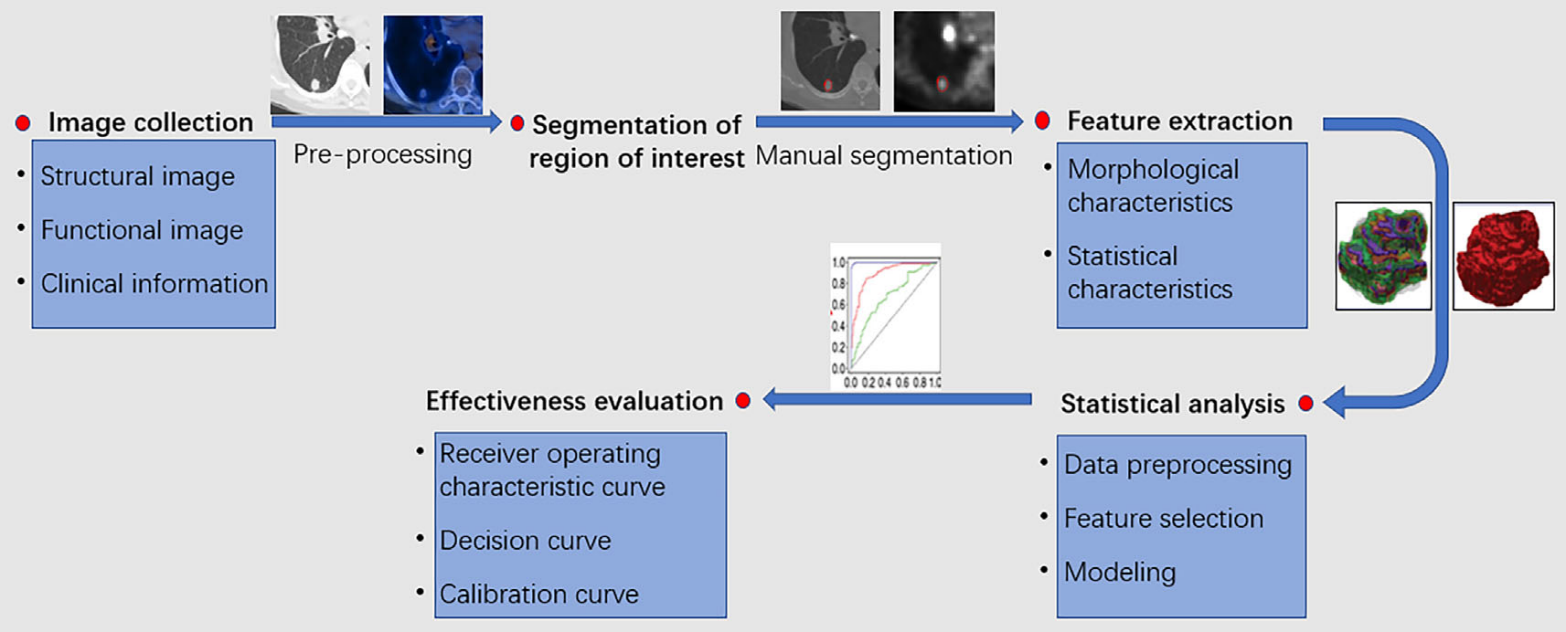
Flow chart of processes for radiomics.

In recent years, methods of omics in the field of radiology have matured. Radiomics can provide more and better information than visual assessment by a clinician. Radiomics refers to the use of automated high‐throughput feature extraction algorithms from radiographic images to convert image data into high‐resolution and discoverable feature space data.^9^ This allows for a comprehensive analysis of tumor phenotypes and ultimately automatic quantitative imaging features that can noninvasively predict nodule and tumor behavior.^10^ The process of assessment with radiomics is shown in Figure 1. Quantitative features associated with disease incidence are analyzed in sub‐images, and visual characteristics provide unique potential for lung cancer screening.^11^

In this study, CT alone, PET alone, and joint PET/CT modeling were used to explore the effect of distinguishing benign and malignant SPNs. The purpose of this study was to compare the value of each model in the differential diagnosis of benign and malignant SPNs.

## 2 METHODS

### 2.1 Patients

**FIGURE 2:**
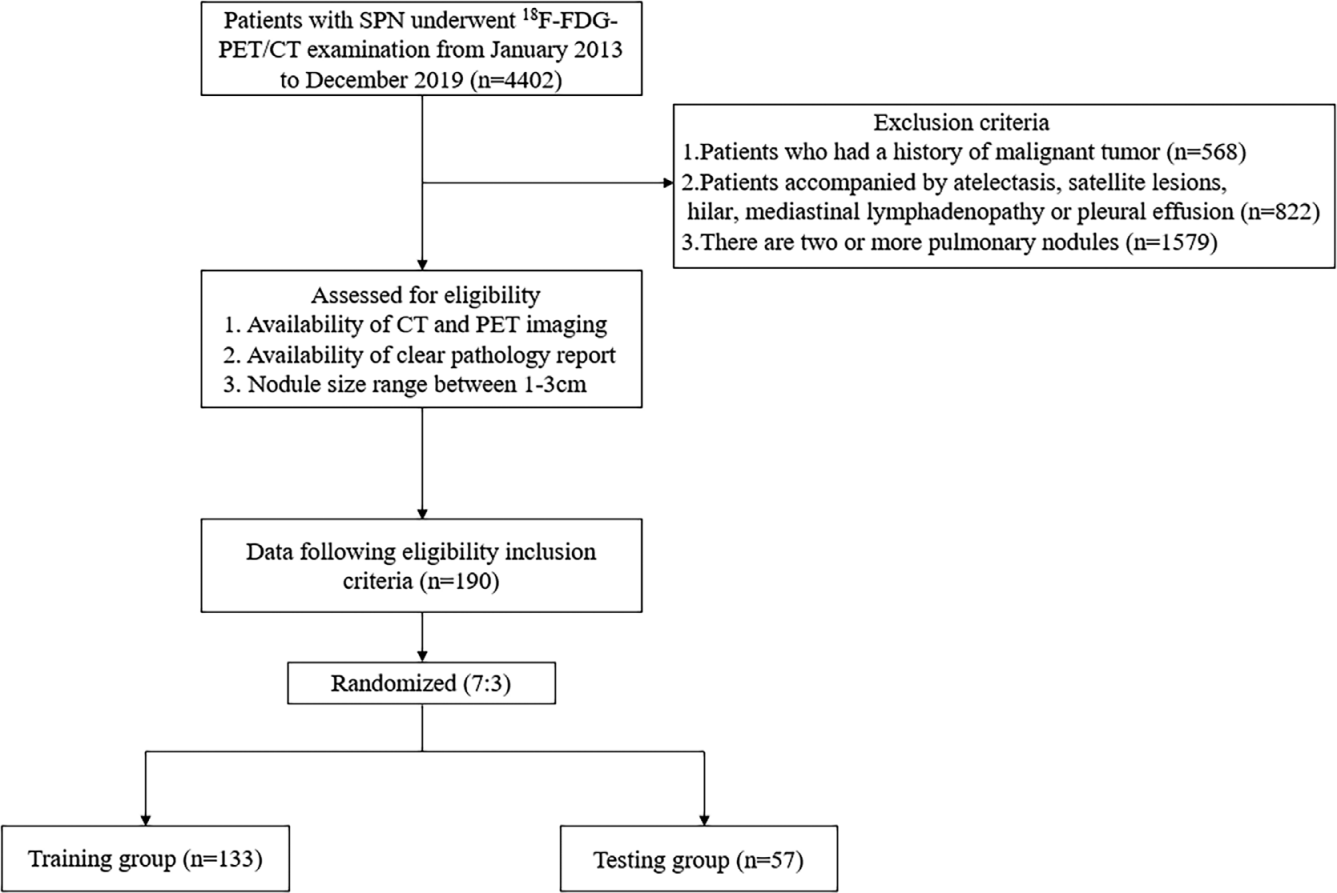
Flow chart for patient enrollment and study design.

A total of 190 patients with SPN who underwent ^18^F‐FDG‐PET/CT examination at our hospital were retrospectively enrolled from January 2013 to December 2019. SPN includes solid and subsolid nodules. Cytologically or histologically confirmation of the final diagnosis of the SPN. A flow chart of patient enrollment and study design is shown in Figure 2. All cases in the training group were used to train the prediction model, while the cases in the independent testing group were used to evaluate the performance of the model.

### 2.2 Imaging

A GE Discovery Elite PET/CT scanner was used, whereby ^18^F‐FDG is produced by a GE Mini Tracer cyclotron and synthesized by an automatic synthesis module, with radiochemical purity >99%. Before examinations, patients fasted for more than 6 h, and the blood glucose was <15 mg/L. The ^18^F‐FDG injection dose was 3.7 MBq/kg body mass, and the patient was examined by routine PET/CT after 60 min of rest. CT scans were taken first with a tube voltage of 120 kV, automatic tube current (15 ~ 180 mA), tube rotation speed of 0.8 s/rot. The original voxel size is 3.65 mm × 3.65 mm × 3.27 mm. PET scans were conducted in 3‐dimensional mode, matrix of 192 × 192, 2 min/bed. The original voxel size is 0.98 mm × 0.98 mm × 3.27 mm. The scanning range covered from the upper part of both thighs to the top of the head. After scanning, the ordered subsets maximum expectation method iteration was used for image reconstruction. PET and CT images were then transferred to a Xeleris workstation for image fusion.

### 2.3 Image segmentation and feature extraction

Digital Imaging and Communications in Medicine (DICOM) format of CT and PET images were imported into the Artificial Intelligence Kit (AK, version 3.3.0, GE Healthcare, China) platform. Both CT and PET images were resampled through linear interpolation to ensure that the voxel was isotropic, with a voxel size of 1.0 mm × 1.0 mm × 1.0 mm. Resampled images were then imported to ITK‐SNAP software (http://www.itksnap.org, version 3.6.0) for segmentation. The three‐dimension (3D) regions of interest (ROIs) were manually delineated along the edges of lesions on all continuous slices on CT images and PET images, respectively. Figure S1 and Figure S2 show original CT and PET images and three‐dimensional images of the ROI in a benign and a malignant case, respectively.

CT and PET images with respective sketched ROI files were imported into the AK platform for radiomics feature extraction. In addition, intraclass correlation coefficients (ICCs) were used to assess the intra‐ and interobserver reproducibility of radiomics feature extraction. To assess interobserver reproducibility, the VOI segmentation of 30 randomly chosen images was performed by two chest radiologists (reader 1 and 2) independently who were blinded to all patients' information. To evaluate internal observer reproducibility, reader 1 repeated the same procedure at a 1‐month interval. Reader 1 completed the remaining image segmentations. Features with ICCs greater than 0.75 indicated good reproducibility and were selected for subsequent analysis.

The maximum SUV of SPNs was measured. The use PET VCAR software to automatically select the entire tumor area as the volume of interest (Volume of Interesting, VOI), and measure the maximum SUV of the primary tumor (SUVmax).

### 2.4 Feature selection and modeling

The synthetic minority oversampling technique (SMOTE) method^12^ was used for sample equalization to produce a benign: malignant ratio of 1:1. Before analysis, outlier and missing values in the training group were replaced by the median. The least absolute shrinkage and selection operator (LASSO) method^13^ with five‐fold cross validation algorithm was then used for dimensionality reduction. This was followed by the Spearman correlation analysis method to remove redundancy, whereby features that correlated highly (|r| > 0.9) with other features were eliminated. Finally, the most meaningful features based on CT and PET images were used for subsequent modeling, respectively. In the training group, the CT radiomics features, PET radiomics features and combined CT and PET radiomics features were used as independent variables with the pathological results of each patient's SPN as the dependent variable. The backward stepwise elimination method was used to construct the multivariate logistic regression model, calculated as: Radiomics signature = β0 + β1 × x1 + β2 × x2 + … … + βn × xn, where β0 is a constant term, xn = {xi, i = 1, 2, …, n} represents the selected radiomics feature, and βn = {βi, i = 1, 2, …, n} represents the feature regression coefficient. Based on this calculation formula, the CT radiomics signatures, the PET radiomics signatures, and the joint radiomics signatures were constructed in the training group.

### 2.5 Performance evaluation

Receiver operating characteristic (ROC) curves, calibration curves, and decision curves were plotted to evaluate the discriminative performance, the calibration degree, and clinical usefulness.

### 2.6 Discrimination

The optimal diagnostic threshold was calculated based on the principle of the maximum Youden index, and then substituted into the independent testing dataset., The area under the ROC curve (AUC), the sensitivity (SEN), specificity (SPE), positive predictive values (PPV) negative predictive value (NPV), and accuracy (ACC) were calculated from ROC analysis in both the training and testing groups to evaluate the diagnostic efficacy of the three models. Besides, the ROC curve of the extracted SUV value was plotted. Delong tests were used to compare whether the difference between AUCs of each radiomics model was statistically significant.

### 2.7 Calibration

Calibration Curves were plotted in both training and testing cohorts to explore the agreement between the observed outcome frequencies and predicted probabilities of the model. The Hosmer–Lemeshow test was used to determine the goodness of fit of the models, and *p* values of more than 0.05 were considered well‐calibrated.

### 2.8 Clinical usefulness

Decision curve analysis (DCA) was performed to evaluate the net benefit for clinical application of the model by quantifying the net benefits at different threshold probabilities.

### 2.9 Statistical analysis

The baseline characteristics and SUV_max_ value in the training and testing cohorts were compared using Student's t‐test or the Mann–Whitney U‐test for continuous variables and the chi‐squared test or Fisher's exact test for categorical variables. All statistical analyses were performed using R (version 3.5.1). The “glmnet”, “pROC”, “rms” and “rmda” packages in R were used in this study. A two‐tailed *p* < 0.05 indicated a statistical significance.

## 3 RESULTS

### 3.1 Baseline characteristics

The average age of the 190 patients with SPNs was 59.7 ± 9.3 (33–80) years, and the sample included 99 males. There were 69 patients confirmed to be benign and 121 confirmed to be malignant. There were 132 cases in the training group (48 benign) and 58 cases in the testing group (21 benign). After using the SMOTE method for equalization treatment, there were 168 cases in the training group (84 benign) and 74 cases in the testing group (37 benign). Patient characteristics and statistical analyses are summarized in Table 1. There were no significant differences between the training and testing cohorts in terms of sex and ages (all *p* > 0.05).

**TABLE 1:** Patient characteristics and the distribution of the radiomics scores.

Variable	Training (N = 132)				Testing (N = 58)			
	Benign (N = 48)	Malignant (N = 84)	Statistics	*p* value	Benign (N = 21)	Malignant (N = 37)	Statistics	*p* value
Sex, Male (%)	28 (58.3)	38 (45.2)	2.100	0.148	15 (71.4)	15 (40.5)	5.120	0.024*
Age, Median (25%, 75%) years	56.79 (36, 80)	60.15 (33, 81)	1.977	0.736	57.57 (35, 77)	62.11 (42, 72)	1.870	0.343
CT score	−0.71 (−1.59, −0.23)	0.52 (−0.78, 1.35)	−4.447	<0.001*	−0.65 (−1.76, 0.37)	1.39 (0.15, 2.24)	−4.571	<0.001*
PET score	−0.78 (−1.71, −0.00)	0.77 (−0.48, 1.99)	−5.510	<0.001*	−0.65 (−1.41, 0.06)	0.81 (−0.29, 1.57)	−3.066	<0.001*
Integrated score	2.35 (−4.08, −0.43)	2.19 (0.45, 4.08)	−7.928	<0.001*	−1.68 (−4.05, 0.72)	1.54 (1.06, 3.33)	−4.457	<0.001*

* *p* value of < 0.05, indicating a significant difference between the two groups. Where appropriate, Chi‐Square tests were used to compare differences in categorical variables, while independent sample t‐tests were used to compare differences in continuous variables. CT; computed tomography, PET; positron emission tomography.

### 3.2 Establishment of the radiomics models

A total of 396 radiomics features were automatically generated from CT and PET images, respectively, and composed of four categories: first‐order histogram features (n = 42, provided the spatial distribution of multiple voxel values), shape features (n = 9, 3D shape related features), second‐order texture features (n = 345, provided the heterogeneity differences via a density histogram and the relative spatial locations of pixels, including 144 Gy level co‐occurrence matrix features (GLCM) with an offset of 1/4/7, 180 Gy level run length matrix (GLRLM) features with an offset of 1/4/7, 11 Gy level size zone matrix (GLSZM) features and 10 haralick features). Details of radiomics features are described in Supporting Figure S3. The image biomarker standardization initiative (IBSI) was regarded as reference and taken into consideration in most of the data processing and features extraction procedure.

The intraclass correlation coefficient (ICC) results are shown in Figure S4. The dimensionality reduction of the individual CT images, individual PET images, and joint image data from the LASSO method (Figure S5) produced 17, 29, and 25 coefficients with non‐zero features, and the number of remaining features after Spearman correlation de‐redundancy was 16, 19, and 25, respectively. The calculation formulas of the models based on the above radiomics features are shown in Supplemental Methods. The distribution of the three radiomics scores were as shown in Table 1, which were all significantly different between the benign and malignant SPNs in both training and testing cohorts.

### 3.3 Performance of the radiomics models

**FIGURE 3:**
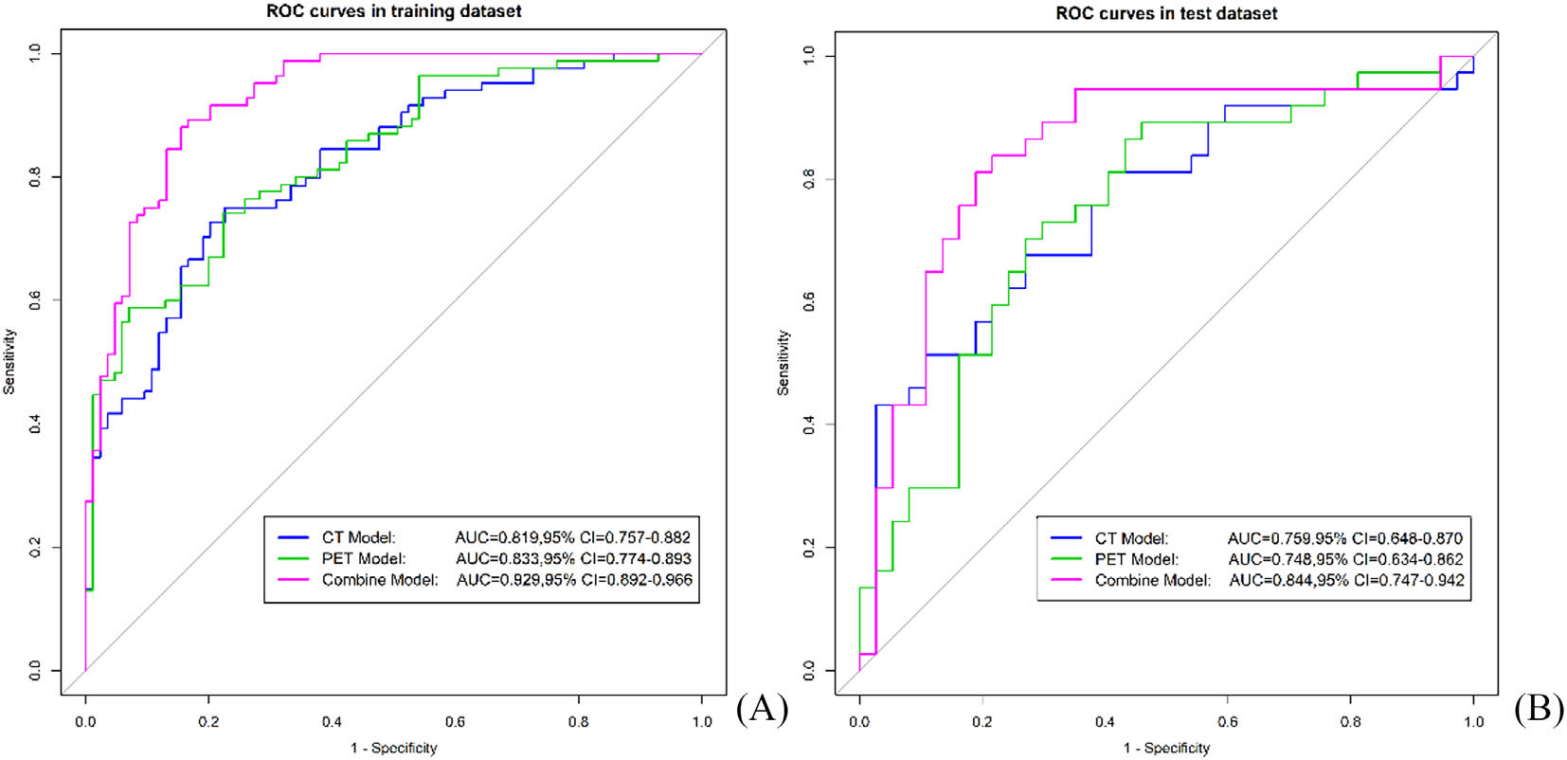
ROC curves of the CT, PET and joint radiomics model in the training (A) and testing (B) groups. AUC; area under curve, CT; computed tomography, PET; positron emission tomography, ROC; receiver operating characteristic.

**FIGURE 4:**
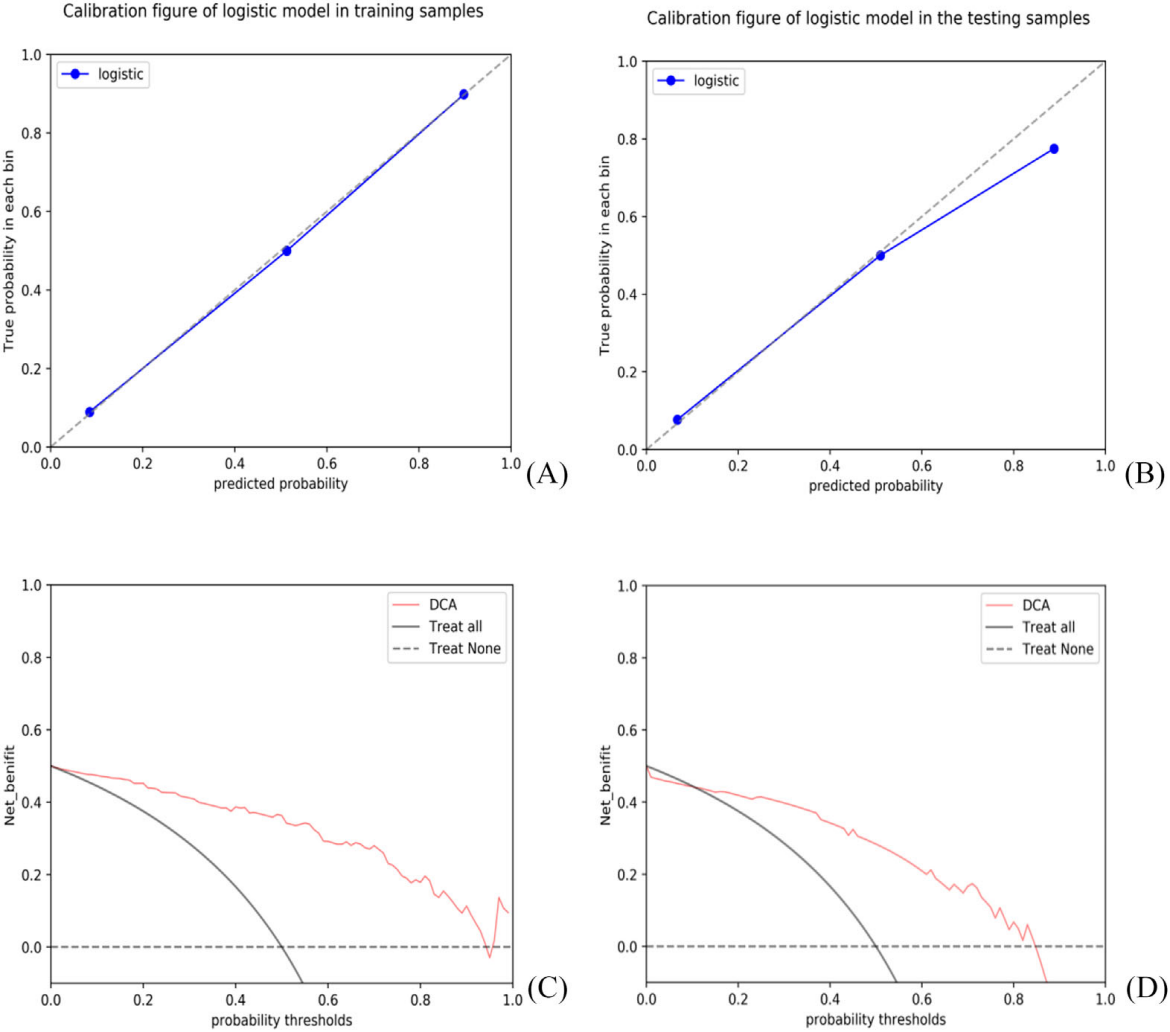
Calibration curves for the joint model in the training (A) and testing group (B), and decision curves for the joint model in the training (C) and testing group (D). DCA, decision curve analysis.

ROC curves of each model are shown in Figure 3. The calibration curve and decision curve of the joint model are shown in Figure 4.

In the training and testing group, the predicted the benign/malignant SPN AUC values were 0.821 and 0.768 in the CT model, 0.820 and 0.738 in the PET model, and the ROC curve AUC values were 0.929 and 0.844 in the joint model, respectively. Delong tests showed that there was no statistically significant difference in the diagnostic efficacy of the three prediction models between the training group and the testing group (*p* > 0.05). The SPE, SEN, and ACC of the joint prediction model were 0.845, 0.881, and 0.863, respectively, in the training group, and 0.703, 0.865, and 0.784, respectively, in the testing group (Table 2).

**TABLE 2:** Evaluation efficiency of radiomics model in testing and training groups.

Classification	Joint model		CT model		PET model	
	Train	Test	Train	Test	Train	Test
Accuracy	0.863	0.784	0.756	0.649	0.747	0.703
AUC	0.929	0.844	0.819	0.759	0.833	0.748
Sensitivity	0.881	0.865	0.702	0.676	0.741	0.649
Specificity	0.845	0.703	0.810	0.622	0.753	0.757
PPV	0.851	0.744	0.787	0.641	0.750	0.727
NPV	0.877	0.839	0.731	0.657	0.744	0.683

AUC; area under curve, CT; computed tomography, PET; positron emission tomography, PPV; positive predictive values, NPV; negative predictive value.

**FIGURE 5:**
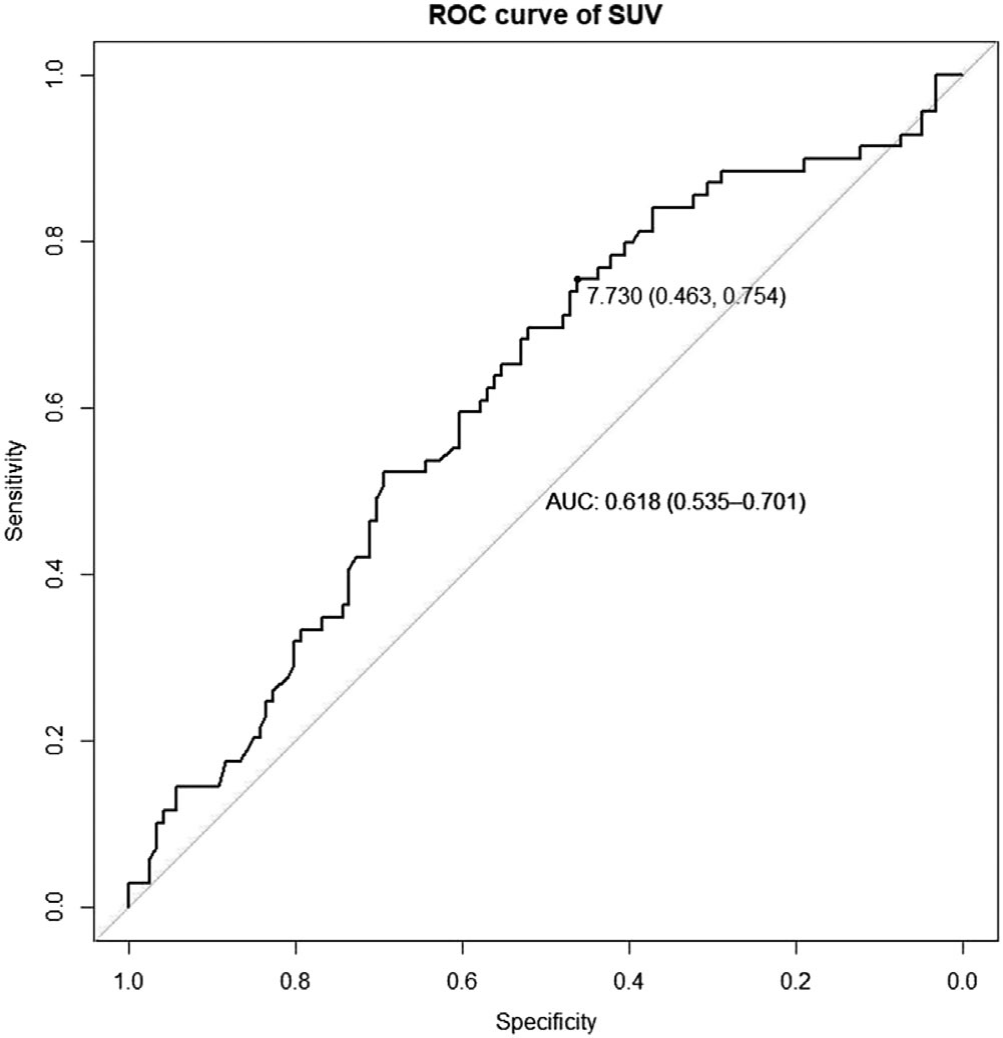
ROC curve of SUV. AUC; area under curve, ROC; receiver operating characteristic, SUV; standardized uptake value.

In addition, we measured and extracted the ROC curve for the SUV from each case. The AUC, SPE, and SEN values were 0.618, 0.463 and 0.754, respectively (Figure 5).

## 4 DISCUSSION

The primary aims of our study were to evaluate the ability of PET, CT and combined PET/CT based radiomics to predict benign and malignant SPNs. The performance of the joint model was better than that of the single model and had a lower SPN misdiagnosis rate, proving its utility for predicting benign and malignant SPNs. In addition, the calibration and decision curves in this study have a good fit, which has value for clinical applications. PET and CT radiomics features were shown to be complementary in the characterization of pulmonary lesions. Noninvasive and cost‐effective radiomics data can help to distinguish between benign and malignant SPNs, making it easier to design the subsequent treatment plan.

18F‐FDG‐PET/CT can show the morphological characteristics of nodules, also provides information about glucose metabolism at the molecular level.^14^ Combining CT and PET (PET/CT) can improve the accuracy of the diagnosis of nodules.^15^ However, some studies have shown that the value of 18F‐FDG‐PET/CT in the evaluation of SPNs is overestimated,^16^, ^17^ and its sensitivity for the diagnosis of SPNs does not exceed 70%.^18^ This may be because some lesions with poor FDG activity, such as bronchoalveolar carcinoma and neuroendocrine tumor, can produce false negative results.^2^ Yap et al. showed that the sensitivity of PET for patients with simple bronchioloalveolar carcinoma was only 33%.^19^ Important variables to consider for evaluation of the diagnostic performance of PET are the metabolic behaviors of different tumors. It is worth noting that the SUVmax values of the quantitative features that we extracted from ^18^F‐FDG‐PET/CT are not very satisfactory, resulting in a high false‐positive rate. The SUVmax value represents the “intensity feature of the voxel” and is not dependent on their relationship with neighboring voxels. This measurement method is too crude to reflect possible heterogeneity of the metabolism in the tumor, and therefore, SUVmax cannot be used to objectively and comprehensively describe the characteristics.

The application of radiomics is helpful for the differential diagnosis of SPNs. However, most radiomics research related to lung diseases is based on CT images. There are few which have assessed whether better diagnostic performance can be achieved with PET alone or a combined PET and CT imaging radiomics model. For example, two studies^20^, ^21^ retrospectively analyzed pulmonary nodule patients and performed CT imaging radiomics extraction of SPN characteristics using a logistic regression model to screen the imaging radiomics features and build the model. For the differential diagnosis of benign/malignant nodules, their results showed AUCs of 0.836/0.862 in the training group and 0.809/0.750 in the testing group. The results of our joint model are better. We can speculate that PET data can compensate for CT data to provide better tissue characteristics. Domenico Albano et al.^22^ demonstrated that many RF features of PET play an important role in predicting the properties of SPN, including stable texture features (grey level co‐occurrence matrix, histogram) that are little affected by machine performance, which are included in our study. Barbara Palumbo et al.^23^ confirmed shape and texture features from PET/CT could lead to a better discrimination between benign and malignant SPN compared with standard imaging features alone. This coincides with our conclusion. CT can provide morphological and anatomical information about the tumor, and PET can reflect the metabolic changes occurring in the tumor. PET images taken with ^18^F‐FDG can provide supplementary information for CT image analysis related to the underlying biological processes and be used to establish a PET image imaging radiomics model that can noninvasively explore the state of the entire tumor. Also, since the pixel size is larger in PET images than CT images, the background noise signal is low. A single texture parameter is not enough to describe the overall heterogeneity of tumors, and radiological characteristics of different texture parameters need to be combined to describe tumor lesions.^24^ Combining CT images with PET images to extract high‐quality features can help us to better characterize relevant information in the tumor. The emergence of PET allows gene or protein changes at the micro‐molecular level to be reflected in the macro‐imaging pictures, making noninvasive predictions and assessment of intratumoral heterogeneity possible. The combination of radiomics and PET/CT can provide a more reliable and accurate basis for the diagnosis of SPN, which has the advantages of economy, practicality, objectivity, and efficiency.

In recent years, studies have shown that texture analysis has great potential for predicting the prognosis of lung cancer. Radiomics reveals tissue heterogeneity characteristics that cannot be observed by the human eye, reflects subtle differences between different tissues, and is not affected by subjective analysis or professional level. It connects image features with tumor features so as to provide valuable information for diagnosis and prognosis. Ahn et al.^25^ found that texture feature analysis can be used as a biological indicator to predict the survival of patients with non‐small cell lung cancer (NSCLC) undergoing concurrent chemoradiotherapy. Mattonen et al.^26^ proposed that the judgment of lung cancer recurrence is often affected by local fibrosis after radiotherapy, and that local recurrence can be detected early using radiomics characteristics based on CT images. Moreover, in patients with NSCLC receiving chemotherapy, textural features of tumoral uptake in ^18^F‐FDG‐PET/CT images are associated with response to chemoradiotherapy and survival, demonstrating its predictive and prognostic capability.^27^ A number of studies have shown that texture indicators have great value in predicting the treatment effect and survival of tumors, and have significantly better predictive value than conventional quantitative indicators of PET images. Zhang et al.^28^ established an imaging model for 248 patients with NSCLC by combining the tumor heterogeneity features extracted from conventional PET and CT. The model could effectively distinguish mutant and wild‐type epidermal growth factor receptor (EGFR), and has high predictive value for EGFR mutations. PET metabolomics models are widely used in clinical practice, and have great value for early diagnosis of disease, prediction of prognosis, and evaluation of curative effect.

Our study had several limitations. 1) There were more malignant cases (190 samples) than benign cases in our sample. This may be due to the performance characteristics of ^18^F‐FDG‐PET/CT examinations, which mainly judge the local and distant staging of malignant tumors. This means that patients with SPN with obvious benign characteristics who had already been diagnosed through conventional imaging were less likely to undergo ^18^F‐FDG‐PET/CT. While we applied the SMOTE algorithm in an attempt to correct the sampling bias, some bias likely remained. 2) three‐dimensional ROIs were artificially delineated based on subjective factors with certain errors. While intraclass correlation coefficient was performed to assess consistency of the features, manual sketching was time‐consuming, and efficient automatic segmentation tools need to be developed. Fully automated segmentation methods for further validation would be attractive in the future.^29^ 3) This was a retrospective study, and so did not incorporate clinical features, leading to specific biases. 4) The established radiomics model lacks multi‐center, large‐sample verification. The next step will supplement external validation.

## 5 CONCLUSIONS

In summary, PET/CT radiomics can be used to effectively predict benign and malignant SPNs with diameters between 1 and 3 cm and can provide a basis for decision‐making for accurate diagnosis, individualization, and precise treatment of lung cancer. With the continuous improvement of image feature extraction technologies, higher classification accuracy, which provides a powerful tool for guiding clinical diagnosis, monitoring, or prognosis, can be obtained.

**List of abbreviations**
CT: computed tomography
PET: positron emission tomography
FDG: fluorodeoxyglucose
SPN: Solitary pulmonary nodule percutaneous
ROI: Region of interest
ROC: Receiver operator characteristic
DCA: Decision curve analysis
LASSO: Least absolute shrinkage and selection operator
AUC: area under curve
SEN: sensitivity
SPE: specificity
PPV: positive predictive value
NPV: negative predictive value
ACC: accuracy
SUV: standardized uptake value

## CONSENT FOR PUBLICATION

Written informed consent for publication was obtained from all participants.

## AVAILABILITY OF DATA AND MATERIALS

The data used to support the findings of this study are available from the first author upon request.

## AUTHOR CONTRIBUTIONS

JCZ analyzed and explained the data of the three models, and was the main contributor to writing manuscripts. JCZ and YH collected the cases together. YG for feature extraction and modeling. MD and PYW outline ROI. JX supervises and guides every link. All authors read and approved the final manuscript.

## CONFLICT OF INTEREST STATEMENT

The authors declare that they have no conflict of interests.

## ETHICS STATEMENT

Our study was approved by the ethical committee of the Shengjing Hospital of China Medical University (2022PS992K). The study was performed in accordance with the ethical standards as laid down in the 1964 Declaration of Helsinki.

## Supporting information


**Data S1.** Supporting Information.

## Data Availability

The data that support the findings of this study are available from the first author upon reasonable request.
