# Supplementary material for: An 18F‐FDG‐PET/CT‐based radiomics signature for estimating malignance probability of solitary pulmonary nodule
Source: Clin Respir J. 2024 May 9;18(5):e13751. doi: 10.1111/crj.13751 (PMC11082539; doi:10.1111/crj.13751)
Supplement: Supplementary file 1 — Data S1. Supporting Information. [file CRJ-18-e13751-s001.docx]

**Supplemental Methods**—Calculation formulas for the models

CT score = 0.089 +

0.315* ShortRunEmphasis_AllDirection_offset7_SD+

0.260* MinIntensity+

0.236* Quantile0.025+

0.203* GLCMEnergy_angle45_offset7-

0.013*VoxelValueSum -

0.199* GLCMEntropy_AllDirection_offset1_SD +

0.404* ClusterProminence_AllDirection_offset7_SD+

0.051* LongRunLowGreyLevelEmphasis_angle90_offset1+

0.514*LowIntensitySmallAreaEmphasis+

0.361* ShortRunLowGreyLevelEmphasis_AllDirection_offset4_SD+

0.122* LongRunEmphasis_angle0_offset1+

0.017*LongRunLowGreyLevelEmphasis_angle0_offset4+

0.385* GLCMEnergy_angle0_offset7-

0.209* GLCMEntropy_angle45_offset7+

0.350* LongRunEmphasis_AllDirection_offset1+

0.254*LongRunLowGreyLevelEmphasis_angle0_offset1

PET score =0.089+

0.569* GLCMEnergy_angle0_offset7-

0.351* ShortRunEmphasis_angle90_offset1-

0.372* Variance+

0.123* ClusterProminence_angle90_offset4+

0.845* InverseDifferenceMoment_angle0_offset4-

1.280* ClusterProminence_angle45_offset1-

0.349* LongRunHighGreyLevelEmphasis_AllDirection_offset1+

0.240* InverseDifferenceMoment_angle90_offset1+

1.057* IntensityVariability-

0.181* Inertia_angle0_offset4+

0.534* LongRunHighGreyLevelEmphasis_angle135_offset1+

1.222* ShortRunHighGreyLevelEmphasis_AllDirection_offset1_SD+

0.617* Inertia_angle90_offset4+

0.137* LongRunHighGreyLevelEmphasis_angle0_offset4-

1.091* ClusterProminence_AllDirection_offset1_SD-

0.349* uniformity+

0.112* LongRunHighGreyLevelEmphasis_angle45_offset1+

1.522* Percentile90-

1.381* HaralickCorrelation_AllDirection_offset1_SD

Integrated score =-0.067+

0.356* LongRunEmphasis_angle0_offset4+

0.206* GLCMEnergy_angle90_offset7+

0.558*LowIntensitySmallAreaEmphasis+

0.317*Inertia_AllDirection_offset1_SD+

0.020* LongRunHighGreyLevelEmphasis_angle90_offset1+

0.480* LongRunLowGreyLevelEmphasis_angle135_offset7+

0.687* LongRunHighGreyLevelEmphasis_AllDirection_offset1+

0.030*skewness-

0.803*HaralickCorrelation_AllDirection_offset1_SD-

0.065*VoxelValueSum+

0.150* GLCMEnergy_angle0_offset4+

0.344* ClusterShade_angle90_offset7+

0.616* Percentile85+

1.554*ShortRunHighGreyLevelEmphasis_AllDirection_offset1_SD+

0.068*MinIntensity-

0.443*GLCMEntropy_AllDirection_offset1_SD+

0.520*inverseDifferenceMoment-

0.406* ClusterShade_angle45_offset1+

1.027*ShortRunEmphasis_AllDirection_offset4_SD-

0.972* ClusterProminence_angle90_offset4+

0.533* LongRunHighGreyLevelEmphasis_angle90_offset4+

1.212* ClusterShade_angle90_offset4-

1.081*ClusterProminence_AllDirection_offset1_SD+

0.607* LongRunLowGreyLevelEmphasis_angle90_offset1+

0.376* LongRunEmphasis_angle90_offset1

Features of PET combined with CT radiomics include:LongRunEmphasis_angle0_offset4(P), GLCMEnergy_angle90_offset7(T), LowIntensitySmallAreaEmphasis(T), Inertia_AllDirection_offset1_SD(P), LongRunHighGreyLevelEmphasis_angle90_offset1(P), LongRunLowGreyLevelEmphasis_angle135_offset7(T), LongRunHighGreyLevelEmphasis_AllDirection_offset1(T), skewness(P), HaralickCorrelation_AllDirection_offset1_SD(P), VoxelValueSum(T), GLCMEnergy_angle0_offset4(T), ClusterShade_angle90_offset7(T), Percentile85(T), ShortRunHighGreyLevelEmphasis_AllDirection_offset1_SD(P), MinIntensity(T), GLCMEntropy_AllDirection_offset1_SD(T), inverseDifferenceMoment(P), ClusterShade_angle45_offset1(P), ShortRunEmphasis_AllDirection_offset4_SD(T), ClusterProminence_angle90_offset4(T), LongRunHighGreyLevelEmphasis_angle90_offset4(P), ClusterShade_angle90_offset4(P), ClusterProminence_AllDirection_offset1_SD(P), LongRunLowGreyLevelEmphasis_angle90_offset1(T), LongRunEmphasis_angle90_offset1(T).

(T) indicates that the feature comes from the CT image, (P) indicates that the feature comes from the PET image.

**Supplemental Figures**

**
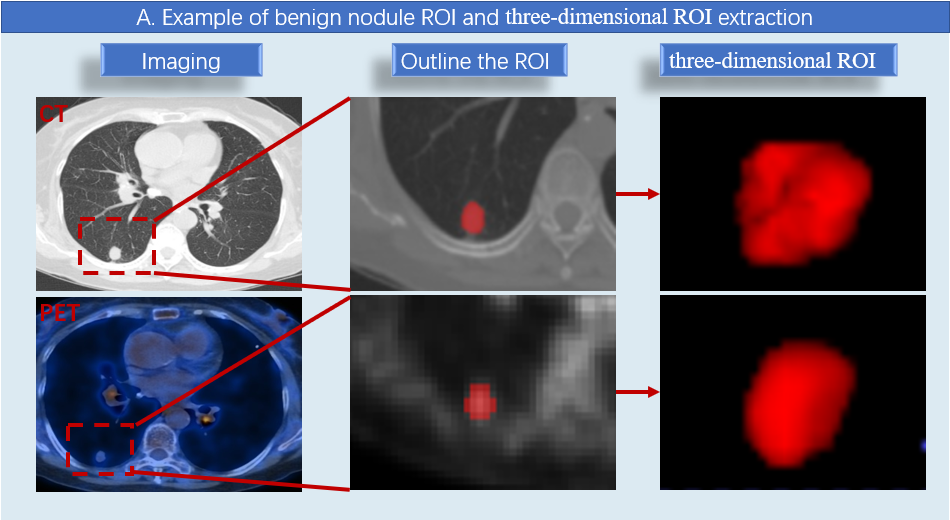
**

**Fig. S1—**Seventy-six-year-old woman with postoperative pathological diagnosis of granulomatous lesions, nodules sized approximately 1.4 cm × 1.2 cm. CT; computed tomography, PET; positron emission tomography, ROI; region of interest.

**
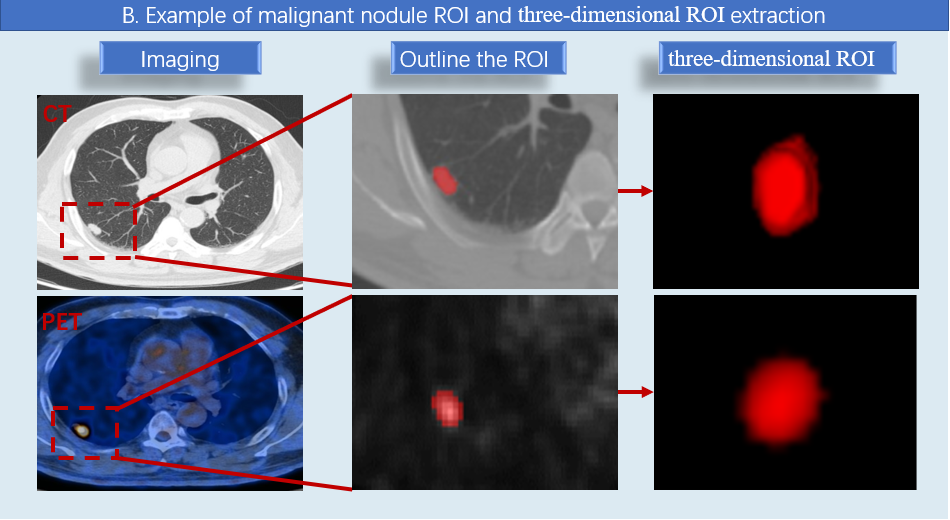
**

**Fig. S2—**Fifty-three-year-old man with postoperative pathological diagnosis of adenosquamous carcinoma, nodules sized approximately: 2.1 cm × 1.0 cm. CT; computed tomography, PET; positron emission tomography, ROI; region of interest.

**
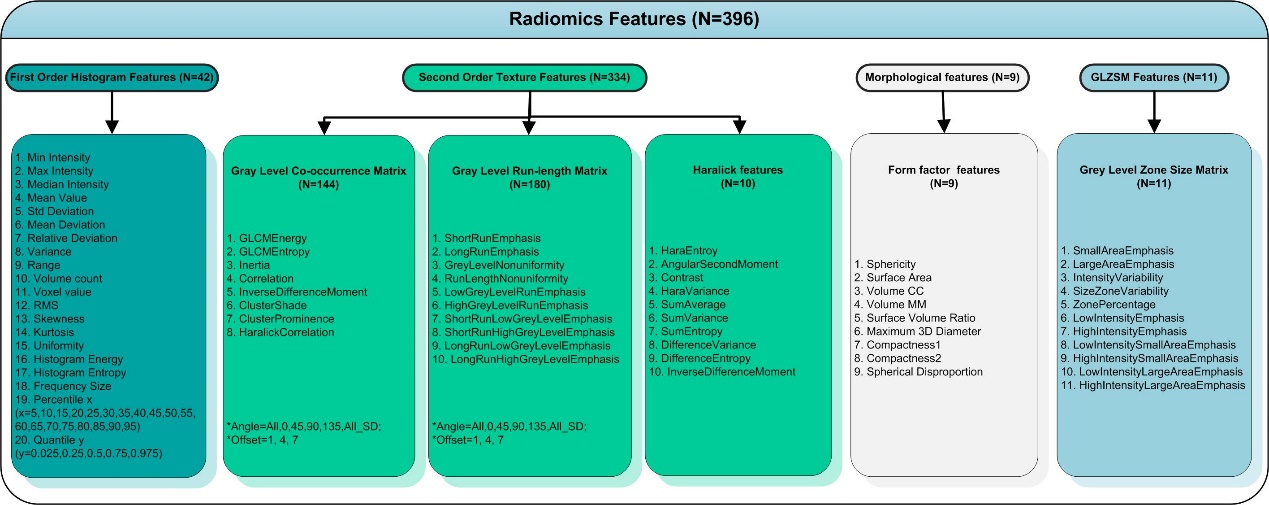
**

**Fig. S3—**Details of radiomics features


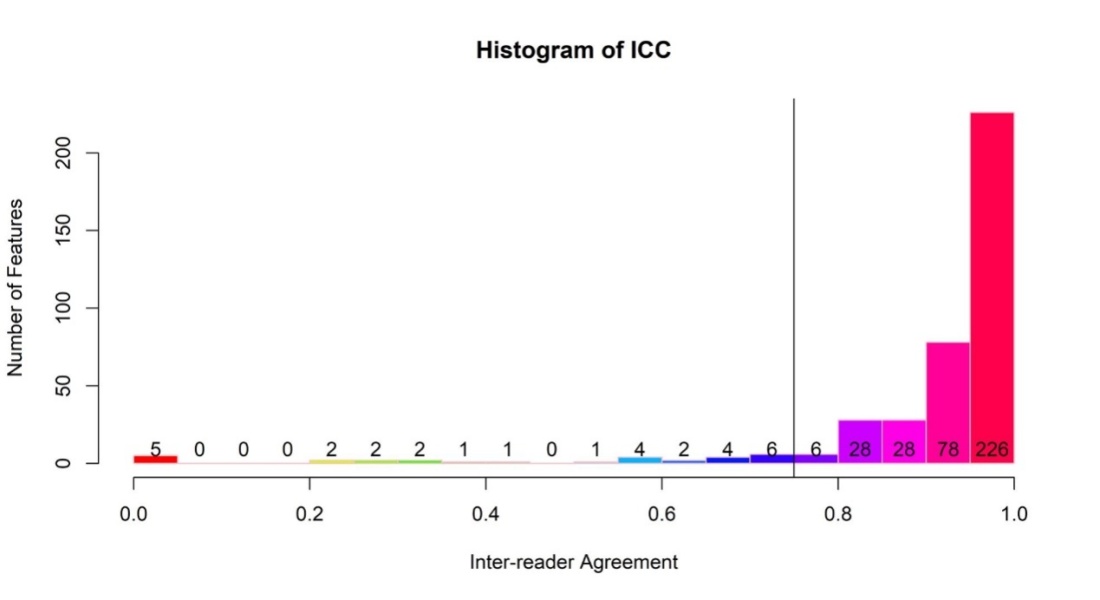

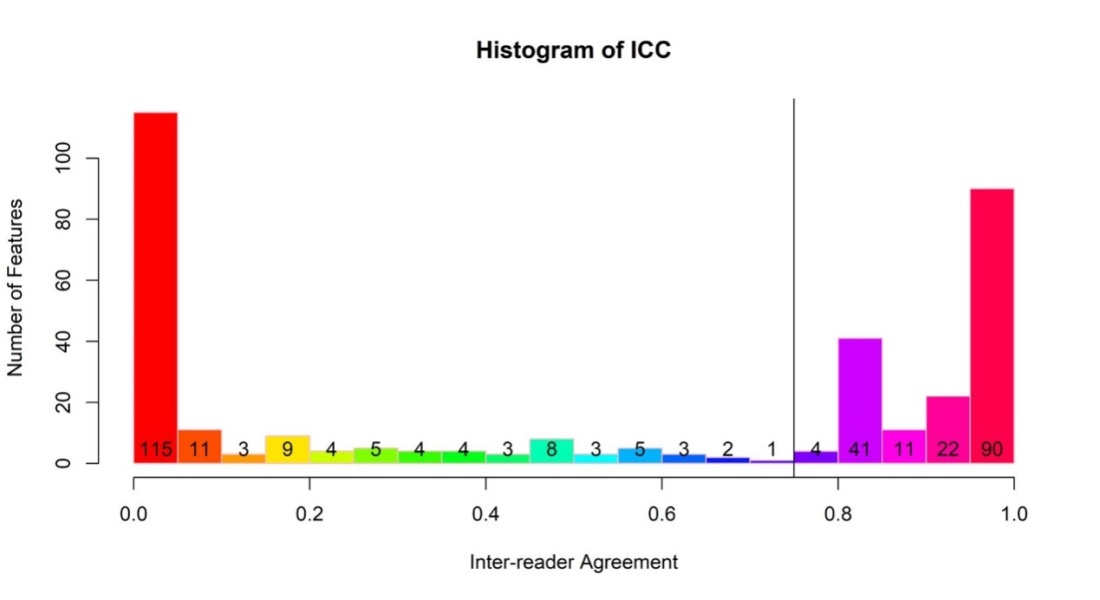


**Fig. S4—**Intraclass Correlation Coefficient (ICC) results.


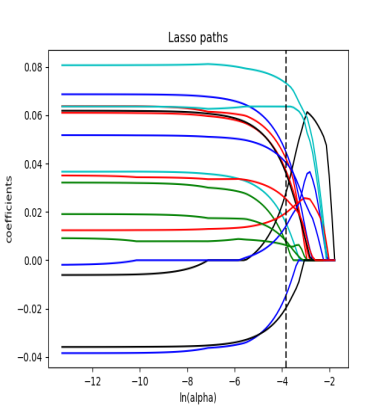

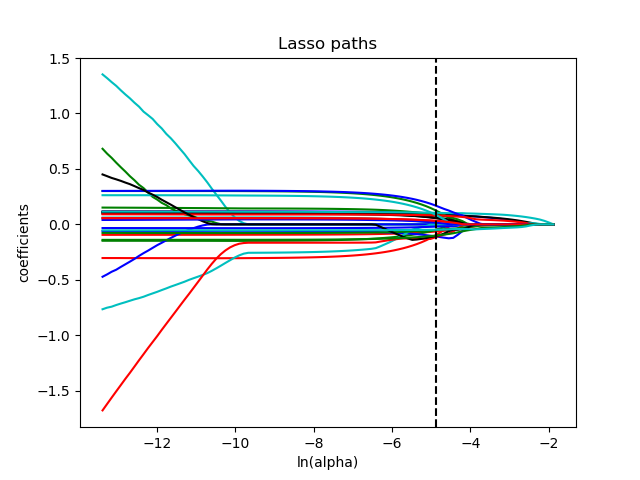

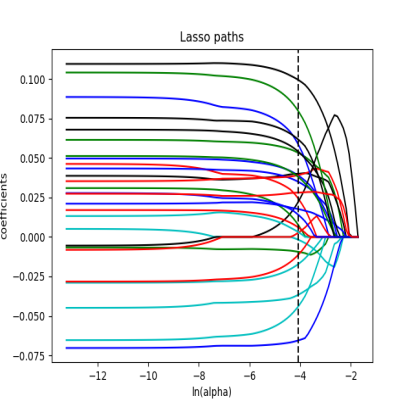


**Fig. S5—**From left to right: LASSO images of CT alone, PET alone, and CT combined with PET. CT; computed tomography, PET; positron emission tomography, LASSO; least absolute shrinkage and selection operator.
